# Selenofuranoside Ameliorates Memory Loss in Alzheimer-Like Sporadic Dementia: AChE Activity, Oxidative Stress, and Inflammation Involvement

**DOI:** 10.1155/2015/976908

**Published:** 2015-05-21

**Authors:** Cristiano Chiapinotto Spiazzi, Melina Bucco Soares, Aryele Pinto Izaguirry, Laura Musacchio Vargas, Mariane Magalhães Zanchi, Natasha Frasson Pavin, Ricardo Ferreira Affeldt, Diogo Seibert Lüdtke, Marina Prigol, Francielli Weber Santos

**Affiliations:** ^1^Laboratório de Biotecnologia da Reprodução (Biotech), Universidade Federal do Pampa (UNIPAMPA), Campus Uruguaiana, 97500-970 Uruguaiana, RS, Brazil; ^2^Instituto de Química, Universidade Federal do Rio Grande do Sul (UFRGS), Avenida Bento Gonçalves 9500, 91501-970 Porto Alegre, RS, Brazil; ^3^Laboratório de Avaliações Farmacológicas e Toxicológicas Aplicadas às Moléculas Bioativas (LaftamBio), Universidade Federal do Pampa (UNIPAMPA), Campus Itaqui, 97.650-000 Itaqui, RS, Brazil

## Abstract

Alzheimer's disease (AD) is becoming more common due to the increase in life expectancy. This study evaluated the effect of selenofuranoside (Se) in an Alzheimer-like sporadic dementia animal model. Male mice were divided into 4 groups: control, A*β*, Se, and A*β* + Se. Single administration of A*β* peptide (fragments 25–35; 3 nmol/3 *μ*L) or distilled water was administered via intracerebroventricular (i.c.v.) injection. Selenofuranoside (5 mg/kg) or vehicle (canola oil) was administered orally 30 min before A*β* and for 7 subsequent days. Memory was tested through the Morris water maze (MWM) and step-down passive-avoidance (SDPA) tests. Antioxidant defenses along with reactive species (RS) were assessed. Inflammatory cytokines levels and AChE activity were measured. SOD activity was inhibited in the A*β* group whereas RS were increased. AChE activity, GSH, and IL-6 levels were increased in the A*β* group. These changes were reflected in impaired cognition and memory loss, observed in both behavioral tests. Se compound was able to protect against memory loss in mice in both behavioral tests. SOD and AChE activities as well as RS and IL-6 levels were also protected by Se administration. Therefore, Se is promising for further studies.

## 1. Introduction

Alzheimer's disease (AD) is a chronic neurodegenerative pathology mainly related to aging and it represents the most common form of dementia [1]. It is characterized by its progressive and slow deterioration in cognition, memory, functional ability, behavior, and mood.

Several mechanisms are involved in AD pathology, such as the presence of neurofibrillary tangles, inflammation processes, and neuronal degeneration. Nevertheless, one of the major characteristics of AD is the presence of senile plaques in brain regions. These plaques are formed by a dense core of aggregated amyloid-*β* (A*β*) peptide. This peptide is formed by proteolytic process of amyloid precursor protein (APP) by the action of *β*-secretase and *γ*-secretase, and it may vary between 39 and 43 amino acid residues [2].

The 25–35 A*β* (A*β*_25–35_) fragment is regarded as the cytotoxic sequence of the parent peptide due to its high level of aggregation, and it retains the toxicity of the total length of the peptide. Possibly, its toxicity may be influenced by the redox state of terminal-C methionine [3], which could be affected by high levels of reactive species.

Furthermore, many studies have demonstrated the relationship between AD and neuroinflammation, which may be a consequence of a systemic inflammatory process that occurs throughout life, mainly related to proinflammatory cytokines such as tumor necrosis factor alpha (TNF-*α*), interleukin 6 (IL-6), and interleukin 1*β* (IL-1*β*) [4]. This process is usually accompanied by an increase in reactive oxygen species (ROS) and oxidative stress.

Therefore, many efforts to treat and prevent the progress of AD have focused in the use of anti-inflammatory and antioxidant compounds. In this scenario, studies on organoselenium compounds have demonstrated both antioxidant and anti-inflammatory properties. Carbohydrate derivatives have emerged as a class of compounds for potential studies, due to its chemical similarity to naturally occurring molecules. Regarding the selenofuranoside compound, a simple carbohydrate that contains a single Se molecule, Vargas et al. [5] demonstrated its effectiveness in restoring *δ*-ALA-D enzyme activity in the ovaries of mice exposed to cadmium. Furthermore, Wollenhaupt et al. [6] recently demonstrated that seleno and telluroxylofuranosides protected against toxicity induced by manganese over the nematode* Caenorhabditis elegans*, leading to nuclear translocation of the transcription factor DAF-16/FOXO, regulating the worms' response to stress, aging, and metabolism. Also, other organoselenium compounds have demonstrated to possess the ability to restore memory loss and cognition impairment induced by A*β* or streptozotocin intracerebroventricular injection [7, 8].

Thus, the aim of this study is to evaluate selenofuranoside, a synthetic organoselenium compound, in a model of Alzheimer-like sporadic dementia induced by intracerebroventricular injection of A*β*_25–35_ in mice, with reference to its possible antioxidant and anti-inflammatory effects and the ability to improve memory loss and impaired cognition.

## 2. Material and Methods

### 2.1. Chemicals

**Figure 1:**
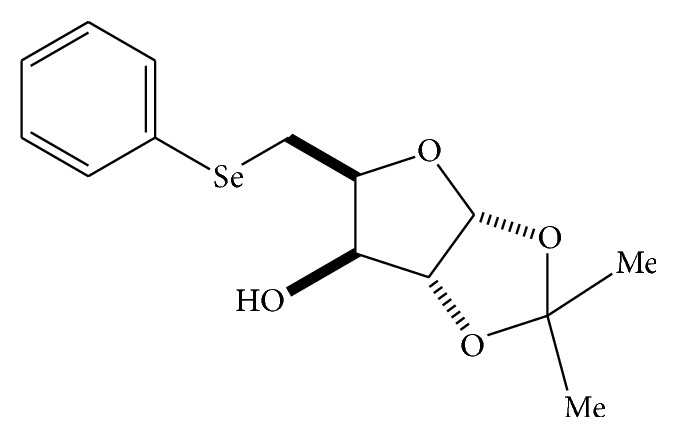
Selenofuranoside structure.

Glutathione reductase, *β*-nicotinamide adenine dinucleotide phosphate reduced tetrasodium salt (NADPH), 5,5′-dithiobis (2-nitrobenzoic acid) (DTNB), reduced glutathione (GSH), glutathione disulfide (GSSG), and amyloid-beta peptide (fragment 25–35) (A*β*_25–35_) were purchased from Sigma-Aldrich (St. Louis, MO, USA). 1-Chloro-2,4-dinitrobenzene (CDNB) was purchased from Aldrich Chemical Co. (USA). Selenofuranoside (Se) was synthesized according to Braga et al. [9] (Figure 1).

The A*β* peptide was dissolved in sterile filtered water and it was aggregated by incubation at 37°C for 4 days before use. Selenofuranoside was dissolved in canola oil.

### 2.2. Animals and Treatments

Adult male Swiss albino mice (25–30 g) were used for this experiment. The animals were kept in an appropriate cabinet for animals with forced air ventilation, in a 12-hour light/dark cycle, at a controlled room temperature of 22°C, with food (Puro Trato, RS, Brazil) and water* ad libitum.* The animals were used according to the guidelines of the Committee on Care and Use of Experimental Animal Resources (Federal University of Santa Maria, Santa Maria, Brazil) and all efforts were made to reduce the number of animals being used and their suffering. This study was approved by the Ethics Committee on the Use of Animals of the Federal University of Pampa (Protocol n° 012/2013).

**Figure 2:**
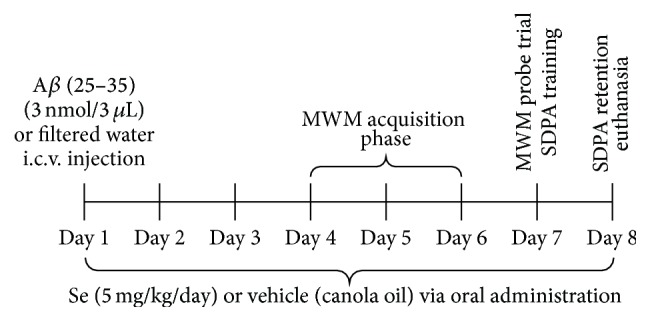
Experimental procedure.

Animals were equally separated into four groups (*n* = 10): control (I); A*β* (II); Se (III); A*β* + Se (IV). Groups III and IV received the Se compound (5 mg/kg/day) orally (intragastric gavage) and groups I and II received vehicle (canola oil) every day, until the end of all behavioral tests (Figure 2).

On the first day of treatment, thirty minutes after Se administration, the mice from groups II and IV received a single administration of A*β* peptide (fragments 25–35) in its aggregated form (3 nmol/3 *μ*L, i.c.v.). Groups I and III received filtered water (3 *μ*L/per site i.c.v.). The dosage of A*β* was based on Wang et al. [10]. Intracerebroventricular injection of the A*β* peptide or vehicle (filtered water) was performed using a stereotaxic (Insight Equipamentos, Brazil) with the bregma fissure as a reference point [11].

Four days after the A*β* peptide (fragments 25–35) injection, the animals have been submitted to behavioral tests. On the eighth day, mice were euthanized and their brains were removed. The whole brains were homogenized in Tris-HCl (50 mM, pH 7.4) and centrifuged. The supernatant (S1) was used for biochemical analysis.

### 2.3. Behavioral Tests

#### 2.3.1. Morris Water Maze (MWM)

Spatial learning and memory were accessed using the MWM task according to Morris [12]. The water maze consisted of a round container (180 cm × 40 cm) made of black plastic and filled with water (22 ± 2°C) at a height of 30 cm. The pool was placed in a room with several visual cues for orientation in the maze. The escape platform was made of the same material and color as the pool and it was placed in the middle of the northwest quadrant, 1 cm below water level. For the acquisition phase, mice were placed next to the wall, facing successively the north, south, east, and west positions. The latency to reach the platform was measured in four trial sessions during 3 consecutive days (corresponding to days 5–7 of treatment) and the animals were allowed to stay in the platform for at least 40 seconds after each trial. Whenever the mice failed to reach the escape platform within 1 min cutoff time, they were retrieved from the pool and placed on it for 40 seconds. Twenty-four hours after the acquisition phase (8th day of treatment), a probe trial was conducted by removing the platform and placing the mice on the north quadrant. The latency as well as the number of crossings over the former platform and the time spent in each quadrant was measured in a single 1-minute trial. The behaviors were videotaped and the experimenter was kept hidden from the animal's sight, but he was able to follow their swimming trajectories.

#### 2.3.2. Step-Down Passive-Avoidance Test (SDPA)

Nonspatial memory was measured using the SDPA task according to Sakaguchi et al. [13] with modifications in the electric shock and exposure time. During the training session (7th day of treatment), each animal was placed on the platform. When it stepped down and placed its four paws on the grid floor, an electric shock (0.5 mA) was delivered for 2 seconds. This procedure was repeated until the animal remained 1 min on the platform. The retention test was performed 24 h after the training. Each animal was placed again on the platform and the step-down latency was measured, considering a cutoff time of 5 min.

#### 2.3.3. Open-Field Test (OPT)

Spontaneous locomotor activity was measured in an open-field test [14] performed on the 8th day of treatment. The floor of the open-field was divided into 9 equal squares. Each animal was placed individually in the center of the arena and the number of segments crossed (4 paws criterion) and rearing was counted in a 4 min session.

### 2.4. Biochemical Analysis

#### 2.4.1. Superoxide Dismutase (SOD) Activity

The activity of SOD was determined as described by Misra and Fridovich [15]. This method is based on the ability of SOD to inhibit the autooxidation of adrenaline to adrenochrome. The color reaction is measured at 480 nm. One unit of enzyme (1 IU) is defined as the amount of enzyme required to inhibit the rate of autooxidation of adrenaline to 50% at 26°C.

#### 2.4.2. Catalase (CAT) Activity

The CAT activity was determined spectrophotometrically according to the method of Aebi [16], which involves monitoring the consumption of H_2_O_2_ in the presence of the sample (S1) (20 *μ*L) at 240 nm. Enzyme activity is expressed in units (1 U decomposes 1 *μ*mol H_2_O_2_/min at pH 7 and 25°C).

#### 2.4.3. Glutathione Peroxidase (GPx) Activity

GPx activity was analyzed spectrophotometrically by the method of Paglia and Valentine [17]. GPx analysis was made by adding GSH, GR, NADPH, and a peroxide to start the reaction, monitored at 340 nm as NADPH is converted to NADP^+^.

#### 2.4.4. Glutathione S-Transferase (GST) Activity

GST activity was analyzed spectrophotometrically at 340 nm, as described by Habig et al. [18]. The reaction mixture contained an aliquot of the homogenized tissue (S1), buffer sodium phosphate 0.1 M pH 7, GSH (100 mM), and 1-chloro-2,4-dinitrobenzene (CDNB) (100 mM), which was used as a substrate. Enzyme activity is expressed as nmol of CDNB conjugated/min/mg protein (30).

#### 2.4.5. Glutathione Reductase (GR)

GR activity in S1 was determined as described by Carlberg and Mannervik [19]. In this assay, GSSG is reduced by GR at the expense of NADPH consumption, which is followed at 340 nm. GR activity is proportional to NADPH decay. The enzymatic activity was expressed as nmol NADPH/min/mg protein.

#### 2.4.6. Glutathione (GSH)

Briefly, an aliquot of S1 was incubated with o-phthalaldehyde (OPT) for 15 min in room temperature [20]. The reaction product was assessed in 420 nm (with excitation in 350 nm). Values were compared to a GSH calibration curve.

#### 2.4.7. Reactive Species (RS)

The levels of the reactive species were determined by a spectrofluorimetric method, using 2′,7′-dichlorofluorescein diacetate (DCHF-DA) assay [21]. An aliquot of S1 was incubated with DCHF-DA (1 mM). The oxidation of DCHF-DA to fluorescent dichlorofluorescein was measured for the detection of intracellular RS. The DCF fluorescence intensity emission was recorded at 520 nm (with 480 nm excitation) 30 min after the addition of DCHF-DA to the medium.

#### 2.4.8. Acetylcholinesterase

For the assessment of AChE activity, samples of the whole brain were homogenized in 0.25 M sucrose buffer (1/10, w/v) and centrifuged at 2400 g at 4°C for 15 min. The activity of AChE was carried out according to the method of Ellman et al. [22], using acetylthiocholine as substrate. The activity of AChE was spectrophotometrically measured at 412 nm. The activity of AChE was expressed as nmol AcSCh/hour/mg protein.

#### 2.4.9. Inflammatory Cytokines

Interleukin-1*β* (IL-1*β*), interleukin-6 (IL-6), and tumor necrosis factor alpha (TNF-*α*) were quantified using ELISA mouse kits for serum and plasma (Sigma-Aldrich, USA).

#### 2.4.10. Protein Determination

Protein concentration was measured by the method of Bradford [23], using bovine serum albumin as a standard.

### 2.5. Statistical Analysis

Data are expressed as mean ± S.E.M. A statistical analysis was performed using a two-way ANOVA followed by the Tukey's test. The main effects are presented only when the interaction effect was nonsignificant. Values of *p* < 0.05 were considered statistically significant.

## 3. Results

### 3.1. Behavioral Tests

#### 3.1.1. Water Maze Test

**Figure 3:**
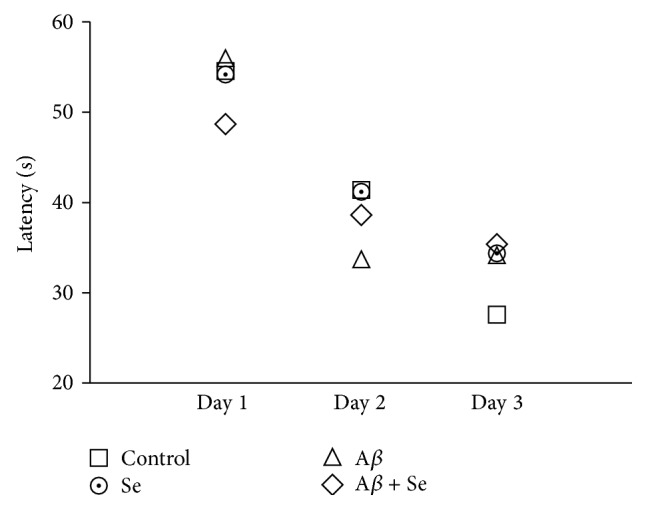
Effect of selenofuranoside on the latency (s) to reach the platform in the acquisition phase in mice with amyloid-*β* induced memory deficit in Morris water maze test (*n* = 10). Data are reported as means.

In the acquisition phase of the WMT, no difference was observed between groups regarding the latency to reach the platform. There was not any type of interaction among treatments or any kind of main effect. However, it is possible to see a significant difference between the days in the acquisition phase in all groups (*F*_1,36_ = 19.40, *p* < 0.001), demonstrating the main effect of time in learning ability (Figure 3).

**Figure 4:**
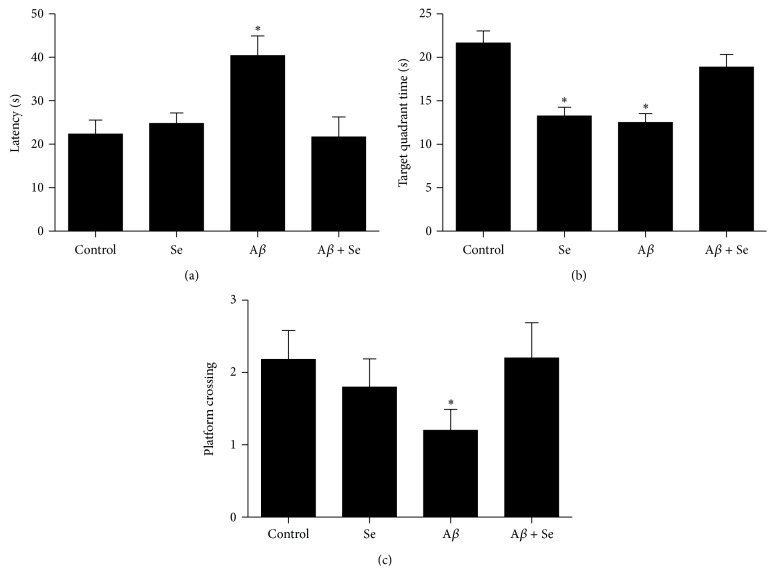
Effect of selenofuranoside on amyloid-*β* induced memory deficit in Morris water maze test (*n* = 10). (a) Latency (s) to reach the platform in the probe test (retention phase). (b) Time (s) spent in the former platform quadrant in the probe test. (c) Number of crossings over the former platform position, in the probe test. Data are reported as means ± S.E.M.  ^*∗*^ *p* < 0.05 as compared to the control group.

In the probe test, a significant increase in latency to reach the former platform for the A*β* group compared to the control group was observed (Figure 4(a); *F*_1,36_ = 4.64, *p* < 0.05). Also, it is possible to identify a difference in the total time spent on the quadrant where the platform was located (Figure 4(b)). The A*β* group presented a significant decrease in this parameter compared to the control group, as well as the Se group. Nevertheless, the Se + A*β* group showed no difference compared to the control group, demonstrating a possible interaction between Se and A*β* (*F*_1,36_ = 18.64, *p* < 0.001).

Two-way ANOVA revealed a significant A*β* × Se interaction (*F*_1,36_ = 5.113, *p* < 0.05) in the number of crossings over the former platform location (Figure 4(c)). Post hoc comparisons showed that the group that received A*β* via i.c.v. presented a significant decrease in the number of crossings compared to the control group, while Se and Se + A*β* groups showed no difference.

#### 3.1.2. Step-Down Passive-Avoidance Test

During the acquisition phase in the passive avoidance, there was no difference in the step-down latency time among groups (*F*_1,36_ = 0.721; *p* < 0.05) (Figure 2). Two-way ANOVA of the step-down latency time in retention trial did not show A*β* × Se interaction (*F*_1,36_ = 2.185; *p* < 0.05), demonstrating a main effect of A*β* (*F*_1,36_ = 12.420; *p* < 0.01).

**Figure 5:**
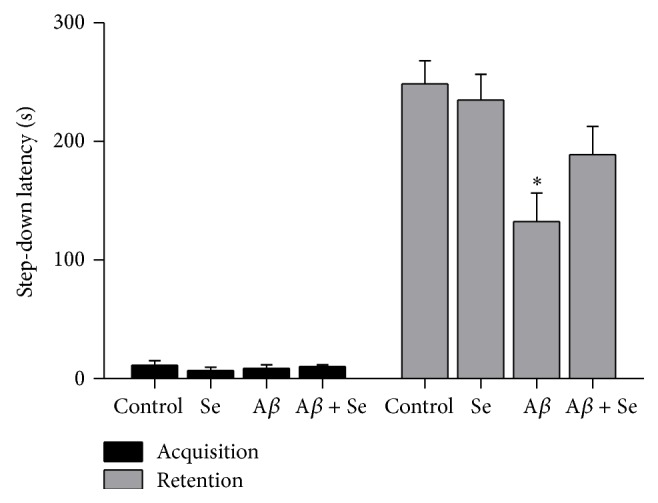
Effect of selenofuranoside on the step-down latency (s) in mice with amyloid-*β* induced memory deficit on the passive-avoidance test (*n* = 10). Data are reported as means ± S.E.M.  ^*∗*^ *p* < 0.05 as compared to the control group.

Post hoc comparisons showed that the A*β* peptide significantly decreased the step-down latency time in retention trial (Figure 5), while Se partially prevented the alteration caused by A*β*.

#### 3.1.3. Open-Field Test

**Figure 6:**
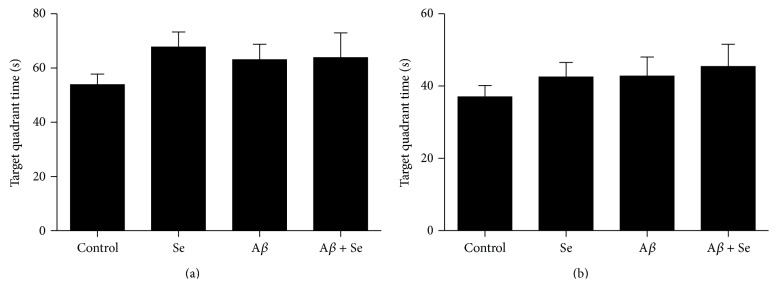
Effect of selenofuranoside in mice with memory deficit induced by amyloid-*β* on the open-field test (*n* = 10). (a) Number of crossings and (b) number of rearings. Data are reported as means ± S.E.M.

The spontaneous locomotor activity measured in the open-field test did not differ significantly between the groups. Two-way ANOVA for the number of crossings (*F*_1,36_ = 1.084; *p* < 0.05) and rearings (*F*_1,36_ = 0.071; *p* < 0.05) revealed no significant differences (Figure 6).

### 3.2. Biochemical Analyses

#### 3.2.1. Oxidative Stress Parameters

Regarding the antioxidant enzymes, an alteration only in superoxide dismutase was observed. Mice that received the A*β* fragment 25–35 via i.c.v. presented a decrease in SOD activity (65%) in brain samples, which was significantly different from the control group (*F*_1,36_ = 19,72, *p* < 0.001). Selenofuranoside therapy was effective to restore enzyme activity at control levels. All the other antioxidant enzymes assessed in this experiment showed no significant alteration (Table 1).

**Figure 7:**
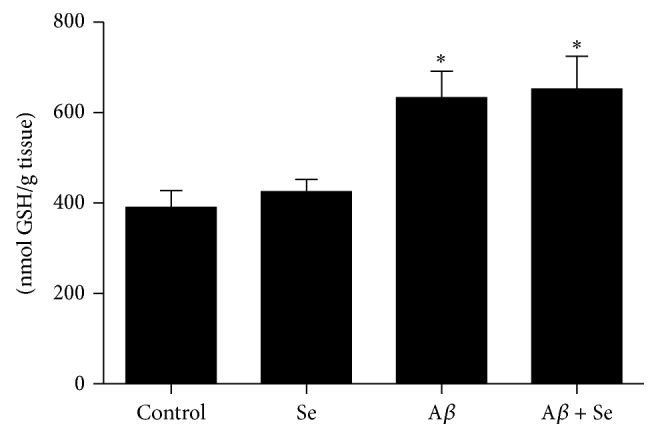
Effect of selenofuranoside on glutathione levels in mice brain after amyloid-*β* treatment (*n* = 10). Data are reported as mean ± S.E.M. and expressed as nmol of GSH per gram of tissue.  ^*∗*^ *p* < 0.05 as compared to the control group.

A main effect of A*β* on GSH levels (*F*_1,36_ = 30.93; *p* < 0.001) was observed in the A*β* group. Increased GSH levels were detected in the A*β* group (40%) as well as in the Se + A*β* group (52%) (Figure 7).

**Figure 8:**
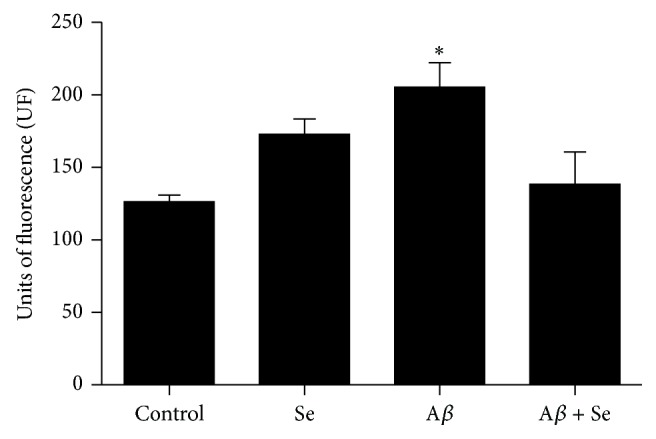
Effect of selenofuranoside on reactive species levels in mice brain after amyloid-*β* treatment (*n* = 10). Data are reported as mean ± S.E.M. and expressed as UF.  ^*∗*^ *p* < 0.05 as compared to the control group.

Two-way ANOVA demonstrated a significant A*β* × Se interaction (*F* = 20.92; *p* < 0.001) in RS levels (55%). An increase in this parameter was found in the A*β* group, compared to the control group (Figure 8). Selenofuranoside was able to reduce RS to the control levels, thus demonstrating a possible protective effect of Se.

#### 3.2.2. AChE Activity

**Figure 9:**
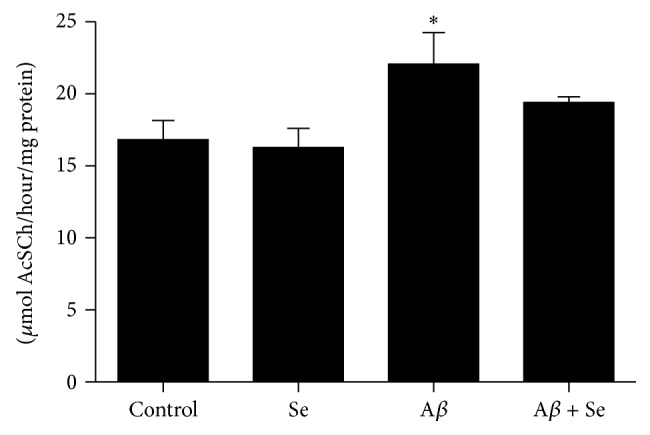
Effect of selenofuranoside on acetylcholinesterase activity in mice brain after amyloid-*β* treatment (*n* = 10). Data are reported as mean ± S.E.M. and expressed as *μ*mol AcSCh/hour/mg protein.  ^*∗*^ *p* < 0.05 as compared to the control group.

The A*β* fragment significantly (*F*_1,36_ = 11.30, *p* < 0.01) increased AChE activity (21%) in the whole mice brain compared to the control group. Selenofuranoside therapy was able to prevent this increase (Figure 9).

#### 3.2.3. Cytokines

**Figure 10:**
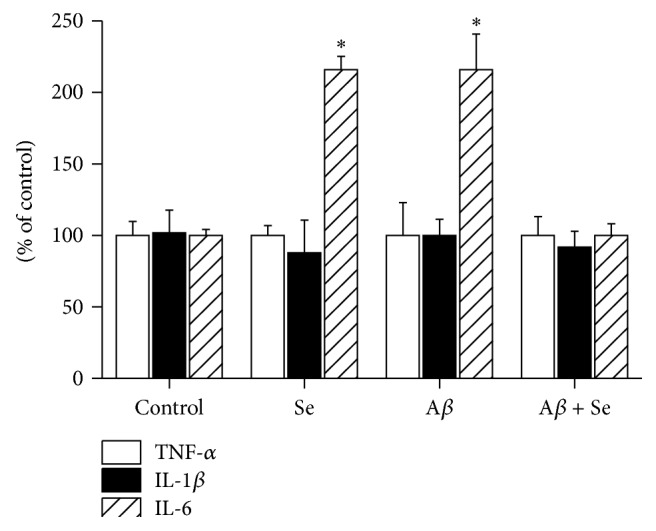
Effect of selenofuranoside on cytokines levels in mice brain after amyloid-*β* treatment (*n* = 3). Data are reported as mean ± S.E.M. and expressed as relative percentage of control.  ^*∗*^ *p* < 0.05 as compared to the control group.

There was a significant A*β* × Se interaction (*F*_1,9_ = 69.65, *p* < 0.001) in IL-6 cytokine (Figure 10). Post hoc tests showed a significant increase in the Se and A*β* groups compared to control. In the Se + A*β* group, IL-6 levels returned to control levels.

IL-1*β* and TNF-*α* showed no significant difference.

## 4. Discussion

In the present study, we evaluated the effect of an organoselenium compound, selenofuranoside, in a model of Alzheimer-like sporadic dementia induced by intracerebroventricular administration of amyloid-beta peptide fragment (25–35). In order to assign new therapeutic approaches, the synthetic antioxidant compounds, such as organoselenium compounds, have been given more attention due to the fact that these compounds present important biological activities.

According to the results, the A*β*_25–35_ i.c.v. injection caused mild memory impairment as described in other papers [7, 24]. This can be concluded by the increased step-down latency in SDPA test and also by the increased latency to reach the former platform location in the MWM test. The A*β*_25–35_ group also showed a reduction in the number of crossings in the former platform location and also in the time spent in the target quadrant during the MWM test. Selenofuranoside was effective to ameliorate most of these evaluated parameters.

Aiming to provide more information related to cognitive impairment and Se intervention, oxidative stress, inflammatory cytokines, and AChE activity were assessed.

Although the role of oxidative stress and reactive species in AD is not clear, many papers have shown that lipid peroxidation and reactive species are increased in an AD brain compared to brains of healthy persons in the same age range, indicating that these increased factors are not merely a consequence of aging [25]. One theory is that methionine-35 of A*β*_(1–42)_ is essential for oxidative stress* in vivo* in AD models and presumably in AD brains as well [26]. Even though the *α*-helix structure of A*β*_(1–42)_ is crucial for the production of oxidative stress [27], apparently the C-terminal Met-35 of A*β*_(25–35)_ fragment also have a strong power to cause mitochondrial dysfunction [28].

The involvement of oxidative stress in memory loss can be seen in our results, since it was possible to see a decrease in SOD activity, one of the most important antioxidant enzymes in most living organisms. This enzyme catalyzes the dismutation of superoxide radical, which is produced as a deleterious byproduct in mitochondrial respiration due to electron leakage. Since mitochondrial dysfunction is regarded as a major causative factor in many neurodegenerative diseases [29], superoxide anion may have accumulated due to the reduction in enzyme activity in the A*β* group. This may be reflected as an increase in reactive species, also seen in the A*β* group. It is likely that a reduction in SOD activity is a primordial event in this process that culminates on reactive species accumulation. These, consecutively, could be responsible for the deleterious effects in the brain.

Selenofuranoside may form selenolate radical (RSe^−1^) in a thiol-rich environment, similarly as diphenyl diselenide and ebselen [30]. Selenolate radical can react with H_2_O_2_ and form selenic acid, which then reacts with thiol group (RSH) to form selenyl sulfide and it can be regenerated back to selenide. This could to some extent explain the reduction in RS levels. However, the most important finding concerning oxidative stress was the ability to restore SOD activity, which probably has most effect on RS levels. Another organoselenium compound, as* p,p*′-methoxyl-diphenyl diselenide, has shown similar effects on SOD activity in different Alzheimer models [31].

Apart from SOD, we did not find significant alterations in other antioxidant enzymes activities, such as GPx, GR, and CAT, nor in xenobiotic detoxification enzyme GST. However, we detected an increase in GSH levels in the A*β* group that was not improved by the selenofuranoside treatment. Since not only GSH is responsible for nonenzymatic oxidative stress balance but it is also used for conjugation reactions to eliminate xenobiotics, the increase seen in the A*β* group may be caused by a response to higher levels of RS or a foreign substance.

Beyond oxidative stress, many studies also indicate an association between inflammation and AD.* In vitro *studies show increased inflammatory cytokines in brain cell cultures submitted to A*β* and the role of microglial activation in AD [32, 33]. Our results showed a significant difference in IL-6 in the A*β* and Se groups compared to control. However, no significant differences were detected in other cytokines evaluated, probably due to different animal model used and/or amyloid-beta fragment. Most* in vivo* studies that show the relationship between inflammatory markers and Alzheimer's disease are conducted on rats or transgenic mice.

A possible involvement of IL-6 on memory processes was observed after knockout mice for this particular cytokine exhibit faster learning skills in the radial maze test through 30 days [34], meaning that this cytokine may be somehow involved in spatial memory. Thus, we verified that animals that received amyloid-*β* presented an increase on IL-6 levels and Se therapy protected this parameter. However, Se itself caused an increase on this parameter. We cannot explain the exact reason for this, and other studies need to be carried out for better understanding of this effect. On the other hand, we believe that the main result in this parameter is related to the combination of amyloid-*β* and Se, in which we verified IL-6 levels similar to the control group.

In addition, many researchers are trying to develop new therapies to treat AD. Acetylcholinesterase inhibitors are still largely used, since it has been hypothesized that cognition impairment may be related to a decrease in acetylcholine levels or an increase in AChE activity [35]. We observed an increase in AChE activity after the A*β* i.c.v. injection, which may explain the results seen in the memory tests employed. Selenofuranoside was able to restore AChE activity as well as memory impairment observed in the A*β* group.

In conclusion, selenofuranoside could be helpful as a new therapeutic research to manage memory loss and impaired cognition since it can modulate AChE activity and protect against reduced SOD activity and increased RS levels after A*β*_25–35_ i.c.v. administration. Also, when administered along with A*β*, selenofuranoside was able to reduce IL-6 concentration to control levels. These effects may be related to the protection against memory loss and cognition impairment presented in behavioral tests. Further studies have to be conducted to evaluate selenofuranoside biological activity as well as its toxicity in order to consider this compound as an alternative therapy for the Alzheimer's disease.

## Figures and Tables

**Table 1:** Effect of selenofuranoside on antioxidant enzymes glutathione peroxidase, glutathione reductase, glutathione S-transferase, catalase, and superoxide dismutase in mice exposed to A*β* (25–35) peptide.

Groups (*n* = 10)	GPx (nmol NADPH/min/mg protein)	GR (nmol conjugated CDNB/min/mg protein)	GST (nmol conjugated CDNB/min/mg protein)	CAT (U/mg protein)	SOD (U/mg protein)
Control	21.38 ± 1.10	24.90 ± 2.15	566.6 ± 26.1	0.628 ± 0.038	51.86 ± 2.10
Se	18.78 ± 1.19	21.96 ± 1.24	707.4 ± 64.8	0.548 ± 0.102	56.36 ± 2.71
A*β*	22.29 ± 1.45	26.93 ± 1.66	653.1 ± 34.6	0.676 ± 0.063	33.85 ± 4.31^∗^
Se + A*β*	19.26 ± 1.26	25.56 ± 2.75	699.7 ± 47.1	0.721 ± 0.083	49.11 ± 1.88

Data are reported as mean ± S.E.M.

^∗^ *p* < 0.05 as compared to the control group.
